# Hook‐shaped enterolith and secondary cachexia in a free‐living grey nurse shark (*Carcharias taurus, Rafinesque 1810)*

**DOI:** 10.1002/vms3.333

**Published:** 2020-08-09

**Authors:** Nicholas M. Otway, Greg J. West, Damian B. Gore, Jane E. Williamson

**Affiliations:** ^1^ NSW Department of Primary Industries Port Stephens Fisheries Institute Taylors Beach NSW Australia; ^2^ Department of Earth and Environmental Sciences Macquarie University Sydney NSW Australia; ^3^ Department of Biological Sciences and MQ Marine Macquarie University Sydney NSW Australia

**Keywords:** CT scan, enterolithiasis, morphometrics, sand tiger shark, X‐ray diffractometry

## Abstract

The carcass of a critically endangered, juvenile female grey nurse shark (*Carcharias taurus*, Rafinesque 1810) was recovered from a south‐eastern Australian beach and subjected to necropsy. The 1.98‐m‐long shark exhibited advanced cachexia with its total weight (19.0 kg) and liver weight (0.37 kg) reduced by 60% and 89%, respectively, compared with a healthy individual of the same length. Marked tissue decomposition was evident preventing histopathology and identification of a definitive cause of death. At necropsy, the abdominal organs were abnormally displaced and showed marked reductions in size compared with a healthy individual of the same size. Importantly, a hook‐shaped enterolith (HSE), with a rough surface and cream in colour, was found within the spiral valve of the intestine and is to the authors’ knowledge, the first description of such in any marine animal. X‐ray diffractometry showed that the HSE comprised the minerals monohydrocalcite (Ca[CO₃].H₂O; ~70 wt%) and struvite (Mg [NH_4_] [PO_4_]. [H_2_O]_6_; ~30 wt%). A CT scan showed concentric lamellate concretions around a 7/o offset J‐hook that formed the nidus of the HSE. Nylon fishing line attached to the hook exited the HSE and was evident in the abdominal cavity through a perforation in the intestinal wall where the posterior intestinal artery merges. The most parsimonious reconstruction of events leading to enterolithiasis and secondary cachexia in this shark was the consumption of a hooked fish and subsequent hook migration causing perforations of the cardiac stomach wall followed by the thin, muscular wall of the apposed, sub‐adjacent intestine.

## Graphical abstract

Hook‐shaped enterolith found in the intestine of a stranded, immature female grey nurse shark, *Carcharias taurus*, that exhibited advanced cachexia. The enterolith was composed of the minerals monohydrocalcite and struvite and a 7/o offset J‐hook formed the nidus. Nylon line attached to the hook (enterolith) was also evident in the abdominal cavity via a perforation of the intestine.

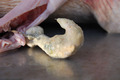

## 1 INTRODUCTION

Enterolithiasis in animals is most common among the equids and has been documented in horses and ponies (Hassel, Aldridge, Drake, & Snyder, 2001), Grant's zebra, *Equus burchelli bohmi*, (Schiffman, 1998) and Hartman's mountain zebra, *Equus zebra hartmannae*, (Decker, Randall, & Prideaux, 1975). These enteroliths are predominantly composed of a concretion of minerals (Blue & Wittkopp, 1981), particularly struvite (Mg [NH_4_] [PO_4_]. [H_2_O]_6_) and their formation has been linked to diet (Hallowell, 2017; Hassel et al., 2001). In other terrestrial animals, enteroliths and/or gastroliths are generally uncommon with most found incidentally at necropsy. Despite this, enteroliths have been documented in domestic cats, *Felis catus*, (Yuki et al., 2006), and also in more exotic species including white‐tailed deer, *Odoocileus virginianus*, (Milton & Axelrod, 1951), the South American tapir, *Tapirus terrestris*, the Malayan tapir, *T. indicus*, (Murphy et al., 1997) and the prehensile‐tailed porcupine, *Coendou prehensilis*, (Spriggs et al., 2014).

Observations of enteroliths and/or uroliths are relatively rare in marine fauna, particularly in free‐living animals and only a few, notable examples have been documented. A 100‐mm spherical, calcium hydroxyapatite enterolith with an octopus beak nidus was surgically removed from the intestine of an adult female sand tiger shark held in London Aquarium (Montreal‐Pawlowsky, Thornton, Stidworthy, & Hale, 2016; Thornton, Monreal‐Pawlowsky, Stidworthy, & Hale, 2012). A carbonate‐apatite/struvite enterolith with a stingray spine nidus was recovered, at necropsy, from a stranded Atlantic bottlenose dolphin, *Tursiops truncatus*, (Burdett & Osborne, 2010). Two struvite/apatite uroliths were found, at necropsy, in the urogenital sinus of a 70‐kg captive, female *C. taurus* from Sea World, Florida USA (Walsh & Murru, 1987). Finally, two 10 mm diameter, enteroliths of unknown composition were also found at necropsy in the distal intestine of a captive green moray eel, *Gymnothorax funebris*, (Boylan et al., 2016). The scant information on enterolithiasis in wild marine animals is especially evident in relation to its occurrence among species, enterolith composition and the causes and clinical consequences. This is likely due to few dedicated programmes undertaking necropsies of marine fauna and the difficulties in finding and accessing carcasses other than cetaceans.

In contrast, accounts of foreign bodies in the gastrointestinal (GI) tract of marine animals are relatively common in the literature and fishing hooks are among the most prevalent items described (e.g. Lécu, Herbert, Coulier, & Murray, 2011; Stoskopf, 1990; Valente et al., 2007, Otway, 2015) as they often have adverse health impacts (e.g. Borucinska, Harshbarger, & Bogicevic, 2003; Borucinska, Kohler, Natanson, & Skomal, 2002; Borucinska, Martin, & Skomal, 2001; Orós, Torrent, Calabuig, & Déniz, 2005). Other foreign bodies reported include stones (Blue & Wittkopp, 1981), ropes and fishing gaffs (Otway, 2015), fishing lines (Franchini et al., 2018) and more recently plastics (Abreo, Blatchley, & Superio, 2019; Gall & Thompson, 2015). In sharks, foreign bodies, especially hooks, can lead to underlying chronic disease and cachexia (e.g. Borucinska et al., 2003; Borucinska et al., 2002; Borucinska et al., 2001) which are characterized by weight loss of muscle and lean body mass (Freeman, 2012, 2018). In advanced cases of cachexia, fat is also lost and in sharks this leads to reduced body and liver weight (Borucinska et al., 2001; Otway, 2015). Apart from weight loss, the effects of cachexia in humans, include anorexia, weakness and poor quality of life, and these symptoms also manifest in other animals. Cachexia also significantly increases morbidity and mortality which cannot be overcome without resolution of the underlying chronic disease (Freeman, 2018).

The grey nurse shark, *Carcharias taurus* (Rafinesque, 1810) is synonymous with the sand tiger and ragged‐tooth sharks from the east coasts of the USA and South Africa, respectively (Last & Stevens, 2009). Off south‐eastern Australia, free‐living *C. taurus* inhabit subtropical to temperate coastal waters and frequently aggregate around rocky reefs at depths of 10–40 m (Otway, Bradshaw, & Harcourt, 2004; Barker, & Williamson, 2010; Otway, & Ellis, 2011; Smith, Scarpaci, Louden, & Otway, 2015). While the shark's maximal longevity exceeds 35 years (Goldman, Branstetter, & Musick, 2006), their late onset of reproduction (10–12 years) combined with low fecundity (two neonates biennially) and minimal genetic variability (Reid‐Anderson, Bilgmann, & Stow, 2019; Stow et al., 2006) means that population recovery from overfishing requires a minimum of decades (Mollet & Cailliet, 2002; Smith, Au, & Show, 1998; Otway et al., 2004). Consequently, *C. taurus* is listed on the International Union for the Conservation of Nature (IUCN) Red List as ‘Critically Endangered’ off south‐eastern Australia and “Vulnerable” globally (Cavanagh, Kyne, Fowler, Musick, & Bennett, 2003). Like many elasmobranchs, they are piscivorous and consume a range of small sharks, rays and teleosts that are swallowed whole (Bass, D’Aubrey, & Kistnasamy, 1975; Lucifora, Garcıa, & Escalante, 2009; Smale, 2005). The species’ penchant for occupying submerged gutters close to emergent rocks can lead to incidental hooking by recreational and/or commercial fishers targeting teleosts (Otway et al., 2004; Bansemer & Bennett, 2010; Robbins, Peddemors, Broadhurst, & Gray, 2013). These interactions can result in sharks being released with hooks in their GI tract causing localized tissue trauma, bacterial infection, chronic pathologies and ultimately death (e.g. Borucinska et al., 2003; Borucinska et al., 2002; Boruscinska et al., 2001; Kneebone, Chisholm, Bernal, & Skomal, 2013; Otway, 2015). Fortuitously, a multi‐faceted, threatened species research programme over the past decade has facilitated detailed necropsies of incidentally captured *C. taurus* and the enumeration of hook‐induced injuries.

This case report documents necropsy results for a stranded, female *C. taurus* recovered from One Mile Beach, Port Stephens, NSW, Australia (32° 46.755’S, 152° 7.010’E) on 5 March 2009. The stranding incident was reported at 1,300 hr by a local council lifeguard and, when recovered an hour later, the shark was extremely emaciated, and decomposition was evident. At necropsy, a hook‐shaped enterolith (HSE) was found in the intestine. We use results from necropsies of healthy *C. taurus* to assess the anatomical changes associated with enterolithiasis and secondary cachexia in this shark. We also describe the structure and composition of the enterolith and the likely events leading to its formation.

## 2 MATERIALS AND METHODS

### 2.1 Necropsies

Necropsies of *C. taurus* commenced by recording total weight (TW, nearest 0.5 kg) using a chain winch with suspended dial‐weighing scale (Salter, Model 235 10X, max 200 kg) rigged on a weighing gantry. While each shark was suspended, a hand‐held metal detector (Ranger Security Detectors Inc., EL Paso, Texas, USA) was used to locate possible retained fishing hooks. Thereafter, carcass lividity, eye condition, colour and presence/absence of gill filaments, tooth colour and their gingival retention, and any spinal deformities were recorded to assess the *ante‐mortem* health and degree of decomposition using criteria modified from Otway (2015). The external examination concluded by measuring 50 morphometric lengths to the nearest mm (e.g. Bass et al., 1975), but are reported to the nearest two decimal places. Included were: total length (TL) with the caudal fin in a depressed position (Francis, 2006); precaudal length (PCL); snout to pectoral fin origin length (SPecO); snout to pelvic fin origin length (SPelO); abdominal cavity length (ACL, via SPelO–SPecO); pectoral‐pelvic space (PPS) and five girths at standardized long‐axis locations (Table 1).

**TABLE 1:** Morphometric relationships derived from healthy free‐living grey nurse sharks (*Carcharias taurus*) subjected to necropsy following capture in the coastal waters off south‐eastern Australia

Relationship	Equation	*n*	*r* ^2^	*F*	*p*
TW on TL	Log_10_TW = 5.4511(Log_10_TL) + 0.7406	390	0.99	31,347.87	<.001
LW on TL	Log_10_LW = 2.8867(Log_10_TL) − 0.3423	60	0.98	3,028.51	<.001
Lengths					
TL on FL	TL = 1.2444(FL) − 0.0204	113	0.99	12,299.79	<.001
TL on PCL	TL = 1.3873(PCL) + 0.0262	113	0.99	11,455.93	<.001
SPecO on TL	SPecO = 0.2426(TL) − 0.0114	113	0.97	3,289.60	<.001
S1DO on TL	S1DO = 0.3904(TL) − 0.0097	113	0.99	22,820.29	<.001
S2DO on TL	S2DO = 0.5959(TL) − 0.0173	113	0.99	27,289.97	<.001
SPelO on TL	SPelO = 0.4992(TL) − 0.0404	113	0.99	16,247.05	<.001
SAL on TL	SAL = 0.6694(TL) − 0.0928	113	0.99	27,292.18	<.001
PPS on TL	PPS = 0.2353(TL) − 0.0699	113	0.89	746.01	<.001
ACL on TL	ACL = 0.2566(TL) − 0.0289	113	0.94	1,796.99	<.001
Girths					
GPecO on TL	GPecO = 0.3372(TL) + 0.1528	113	0.59	167.67	<.001
GPecI on TL	GPecI = 0.3274(TL) + 0.1658	113	0.59	162.98	<.001
G1DO on TL	G1DO = 0.3671(TL) + 0.1676	113	0.58	157.49	<.001
GPelO on TL	GPelO = 0.2980(TL) + 0.1382	113	0.59	167.27	<.001
GPCP on TL	GPCP = 0.1012(TL) + 0.0632	113	0.58	159.57	<.001

Note

Regression for liver weight (LW) on total length (TL) was determined for juvenile females.

Abbreviations: ACL, abdominal cavity length; all lengths and girths in metres; FL, fork length; G1DO, girth at the first dorsal fin origin; GPCP, girth at the cranial edge of the precaudal pit; GPecI, pectoral fin insertion; GPecO, girth at the pectoral fin origin; GPelO, girth at the pelvic fin origin; LW, liver weight in kilogrammes; PCL, precaudal length; PPS, pectoral‐pelvic space; S1DO, length of snout to first (cranial) dorsal fin origin; S2DO, length of snout to second dorsal (caudal) fin origin; SAL, snout to anal fin length; SPecO, length of snout to pectoral fin origin; SPelO, length of snout to pelvic fin origin; TL, total length; TW, total weight in kilogrammes.

**FIGURE 1:**
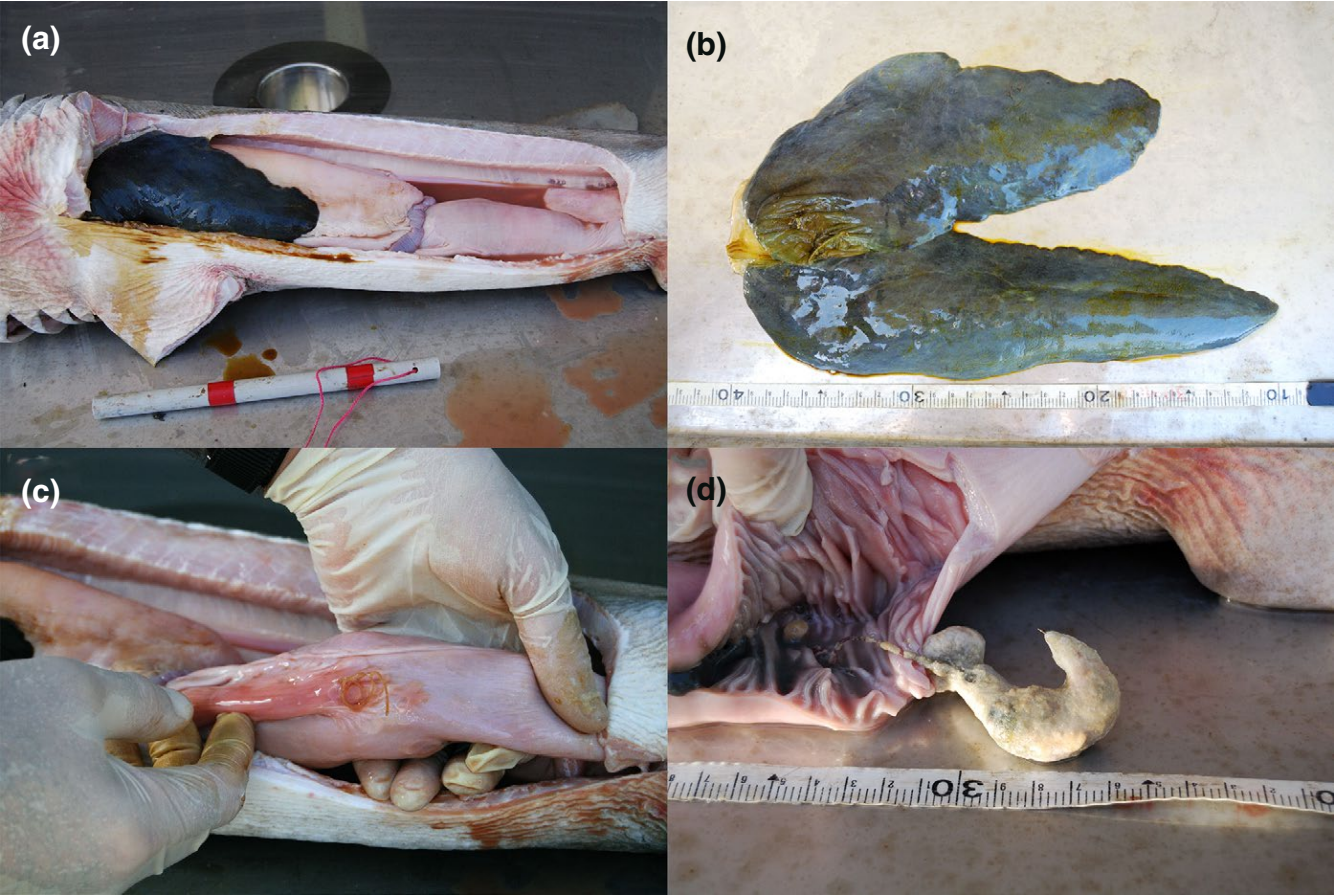
Photographs from necropsy of the stranded, juvenile female grey nurse shark (*Carcharias taurus*) recovered from One Mile Beach, NSW, Australia showing: (a) markedly reduced size of liver and organs of the gastro‐intestinal tract (scale bar = 25 cm); (b) small bluish‐black liver with gall bladder in smaller left lobe (scale: tape with 1 cm increments); (c); orange nylon fishing line protruding through the dorsal wall immediately proximal to the inflamed posterior intestinal artery and (d) intestine with spiral valve and the hook‐shaped enterolith (scale: tape with 1 cm increments)

An internal examination with gross inspection of the organs also included measurement (length and diameter to nearest mm) of the liver, spleen and GI tract organs comprising the descending cardiac stomach, ascending pyloric stomach and intestine with ring/columnar spiral valve (Holmgrem & Nilsson, 1999; Leigh, Papastamatiou, & German, 2017). Tissue samples were taken and preserved in neutral buffered formalin for histopathology. Reproductive status was assessed following standard methods (Gilmore, Dodrill, & Linley, 1983; Walker, 2005) with liver weight (LW) and gonad weight recorded (nearest 0.1 kg). Sexual maturity was evaluated using the TL, PPS, reproductive tract development and presence/absence of uterine hymen. Bile from the gall bladder in the left liver lobe (Figure 1b) was aspirated using a 10 ml disposable syringe and 18G × 38 mm needle (Terumo, Tokyo, Japan). Fluids from the abdominal cavity and organs of the GI tract were aspirated using disposable syringes of varying volumes (10–50 ml). Finally, age was estimated using thin sections of vertebral centra sampled from under the cranial (first) dorsal fin (Goldman et al., 2006).

### 2.2 Laboratory analysis of the HSE

The HSE was dried to constant weight in an air‐tight container with silica gel at 21°C and then weighed (nearest 0.5 g) on an electronic balance. A sub‐sample was pulverized, and elemental composition determined using an Olympus Delta Pro X‐ray fluorescence spectrometer with tantalum anode tube, which can quantify the concentrations of elements from aluminium to uranium. In this study, operating conditions of 50 kV (strontium‐lanthanum), 40 kV (iron‐zinc, arsenic‐rubidium, tantalum‐bismuth) and 15 kV (phosphorus‐manganese) were used, with a total measurement time of 90 s. Limits of quantification vary for each element and sample, but are typically around 0.001 wt% for elements from rows 4 to 6 in the periodic table, and 1–0.1 wt% for elements in row 3. Measurements have typical accuracies of better than 20% where concentrations are >0.1 wt%. Mineralogy was determined using X‐ray diffractometry. A sample (~5 mg) was hand milled under ethanol, dried and mounted as a powder on a silicon crystal low background sample holder. Diffractograms were collected from 5 to 90° 2θ using a PANalytical X'Pert Pro MPD diffractometer, with operating conditions of 45 kV, 40 mA, CuK_α_ radiation, X'Celerator detector, Bragg Brentano geometry and a slew rate of 5° 2Θ per minute. Diffractograms were analysed using PANalytical HighScore + software (Version 2.2.4) with the 2013 International Centre for Diffraction Data Powder Diffraction File 4 inorganic mineralogical database and the FIZ Karlsruhe GmbH Inorganic Crystal Structure Database. The method detection limit is ~0.1 to 2 wt%, depending on the sample matrix and mineral crystallinity.

The size, three‐dimensional shape and internal structure of the HSE was determined using a clinical 5G Cone Beam CT (New Tom^®^) scan at Macquarie Medical Imaging (Macquarie University, NSW, Australia). CT images were visualized using RadiAnt DICOM viewer software (Version 5.01, © Medixant 2019). This also enabled the hook shape and dimensions comprising total length, gape, eye, shank, bite and wire diameter to be estimated (nearest mm) and its overall three‐dimensional position within the HSE to be determined. The weights of 20 new offset J‐hooks of a similar size, shape and wire diameter to the actual hook nidus were then measured (nearest 0.1 g) on an electronic balance and the mean calculated to estimate the weight of the hook within the HSE and, by difference, the dry weight of the mineral component of the HSE.

### 2.3 Statistical analyses

To date, 195 *C. taurus* have been subjected to necropsy following their incidental capture along the south‐eastern Australian coastline and the associated morphometric data from individuals classified as healthy *ante‐mortem* were used to generate least‐squares linear regression relationships. Least‐squares linear regressions of TW and LW on TL were also generated following log_10_ transformation. Differences between the TW, LW, morphometric lengths and girths of healthy *C. taurus* and the stranded female were assessed using 2‐tailed asymmetrical *t*‐tests (Sokal & Rohlf, 1969) with predicted values for a healthy 1.98 m TL individual obtained from each respective linear regression. The dimensions (length, diameter to nearest mm) of the liver lobes, cardiac stomach, pyloric stomach, intestine and spleen were expressed as a proportion of the ACL as this did not change significantly with increasing TL (range, 0.20–0.29). Differences in the proportional lengths of the liver, GI tract organs and spleen between healthy *C. taurus* and the stranded female were assessed using two‐tailed asymmetrical *t*‐tests. To this end, data from a subset of 62 healthy individuals (both sexes) that had not fed for at least 24‐hr *ante‐mortem* were used to control for cardiac stomach expansion that occurs with feeding.

## 3 RESULTS

### 3.1 Healthy *C. taurus*

One hundred and thirteen *C. taurus* (53 males, 60 females) were classified as healthy ante‐mortem and had normal copper‐brown skin and no pallor, eyes normal in appearance, bright red gill filaments and white teeth that were firmly anchored in the cream‐coloured gingiva. None of these sharks had skin lesions, spinal deformities, ingested hooks or other fishing‐related injuries. Histopathological analyses on a subset of these sharks were also unremarkable and confirmed the *ante‐mortem* health assessment. Their TW, LW and morphometric data resulted in significant linear regression relationships accounting for 58%–99% of the variation in the respective dependent variables (Table 1).

### 3.2 Stranded female *C. taurus* with HSE

When recovered from the beach, the stranded female *C. taurus* had minimal lividity remaining, the skin was pale fawn and the normal red‐brown spots had faded substantially. The gill arches and pectoral and pelvic girdles were prominent under the skin because of skeletal muscle loss. The gill filaments were totally absent from the five gill slits on both sides of the shark and its eyes were sunken into the orbits and dehydrated. The orobranchial cavity was abnormally bright white in colour and while the white teeth were anchored within the abnormally white gingiva, they could be removed easily in contrast to recently deceased individuals. There were no spinal deformities or external fishing related injuries, but examination with the metal detector indicated that a likely fishing hook had been retained within the abdominal cavity along the ventral mid‐line, inferior to the cranial (first) dorsal fin origin and cranial to the pelvic fin origins. Finally, tissue samples for histopathology were not collected from the stranded female owing to the degree of *post‐mortem* autolysis.

The results of comparisons between the healthy (*n* = 113) and stranded female *C. taurus* using 2‐tailed asymmetrical *t*‐tests are summarized in Table 2. The 19.0 kg TW of the stranded female *C. taurus* was significantly less than the predicted 47.5 kg of a healthy individual (*p* < .001) and represented a 60% TW reduction. The 1.98 m TL of this female was similar to the predicted 1.99 m TL derived from the estimated age of 4.5 years. The 1.98 m TL was also consistent with the 1.58 m FL and 1.36 m PCL and neither differed significantly from their predicted values (*p* > .05). Similarly, all remaining long‐axis morphometric lengths were not significantly different from respective predicted values (all *p* > .05). The 0.31 m PPS was not significantly different from the predicted value, but was ~42% less than the PPS of sexually mature females (i.e. PPS ≥ 0.53 m at 50% primiparity) and indicative of a sexually immature juvenile. All five girths were significantly less than their respective predicted values (all *p* < .025) and represented ~32%–52% reductions depending on the specific girth. Finally, the 0.47 m ACL in this female was not significantly different from that predicted (*p* > .25).

**TABLE 2:** Comparisons between the cachectic, female grey nurse shark (*Carcharias taurus*) with hook‐shaped enterolith and normal healthy individuals using two‐tailed asymmetrical *t*‐tests based on the morphometric regression relationships in Table 1 with significant (*p* < .05) percent changes bolded

Variable	Comparison of cachectic versus healthy individuals				
	Cachectic	Healthy	% change	*t*	*p*
Weights					
TW_TL_	19.00	47.44	**−59.95**	−12.42	<.001
LW_TL_	0.37	3.27	**−** **88.69**	−19.35	<.001
Lengths					
FL_TL_	1.58	1.61	−1.74	−0.83	>.050
PCL_TL_	1.36	1.41	−3.41	−1.56	>.050
SPecO_TL_	0.48	0.47	1.47	0.31	>.250
S1DO_TL_	0.76	0.76	−0.39	−0.28	>.250
S2DO_TL_	1.17	1.16	0.17	0.14	>.250
SPelO_TL_	0.95	0.95	−0.11	−0.09	>.250
SAL_TL_	1.23	1.23	−0.49	−0.03	>.250
PPS_TL_	0.41	0.40	3.61	0.34	>.500
ACL_TL_	0.47	0.48	−1.46	−0.25	>.250
Girths					
GPecO_TL_	0.55	0.82	**−** **32.93**	−2.26	<.025
GPecI_TL_	0.45	0.81	**−** **44.72**	−3.09	<.005
G1DO_TL_	0.43	0.90	**−** **51.96**	−3.46	<.001
GPelO_TL_	0.37	0.73	**−** **49.16**	−3.39	<.001
GPCP_TL_	0.18	0.26	**−** **31.82**	−2.27	<.025

Note

Lengths and girths in metres; total weight in kilograms; subscript denotes the independent variable in each respective linear regression in Table 1.

Internally, the abdominal cavity contained ~200 ml of serous fluid and the organs of the GI tract were markedly reduced in size (Table 3 for proportional dimensions) and this altered their normal ‘S‐shaped’ position within the abdominal cavity (Figure 1a). The liver was bluish‐black in colour, greatly reduced in size (Figure 1a and b) and the 0.37 kg LW was significantly less than the predicted 3.27 kg (Table 2, *p* < .001) and represented an ~89% reduction. The liver lobes were abnormally different in length and the left lobe, with gall bladder, was ~22% shorter than the right lobe (Figure 1b). The right liver lobe proportional dimensions were significantly less than the respective means in healthy sharks (Table 3, both *p* < .001) and represented reductions of ~39% and ~46% in length and diameter, respectively. Similarly, the left lobe liver proportional dimensions were also significantly less than the respective means in healthy sharks (both *p* < .001) and represented reductions of ~53% and ~38% in length and diameter, respectively. Bile aspirated from the gall bladder (Figure 1b) was a turbid, brown green that contrasted with the clear, bright green coloured fluid in healthy individuals.

**TABLE 3:** Mean (±*SE*) proportional lengths and diameters (of abdominal cavity length) for the liver, GI tract organs and spleen in the cachectic female and *n* = 62 healthy, free‐living grey nurse sharks (*Carcharias taurus*) that had not fed 24‐hr *ante‐mortem* following their capture in the coastal waters off south‐eastern Australia

Organ	Cachectic grey nurse shark		Healthy grey nurse sharks		
	Length	Diameter	*n*	Length	Diameter
Liver					
Right lobe	0.61	0.12	62	0.99 (0.01)	0.22 (0.01)
Left lobe	0.48	0.13	62	1.00 (0.02)	0.21 (0.01)
Cardiac stomach	0.51	0.14	62	0.74 (0.01)	0.22 (0.01)
Pyloric stomach	0.32	0.02	62	0.68 (0.01)	0.03 (0.01)
Intestine	0.27	0.09	62	0.50 (0.02)	0.12 (0.01)
Spleen	0.32	0.01	62	0.67 (0.01)	0.03 (0.01)

The stranded female's cardiac stomach was greatly reduced in size leading to its cranial retraction within the abdominal cavity (Figure 1a). The proportional length and diameter of the cardiac stomach were significantly less than the respective means in healthy *C. taurus* (both *p* < .001) and represented reductions of ~32% and ~38%, respectively. The pyloric stomach was also markedly reduced (~52% and ~32%, respectively) and the proportional length and diameter were significantly less than their respective means in healthy *C. taurus* (both *p* < .001).

The intestine had an irregularly coiled length of orange nylon fishing line (120 mm long × 1.20 mm diameter) protruding through a perforation in the midline of the thin, dorsal wall immediately proximal to the junction of the inflamed, posterior intestinal artery (Figure 1c). Intestinal dimensions were also markedly reduced (~46% and ~26%, respectively) and the proportional length and diameter were significantly less than their respective means in healthy *C. taurus* (both *p* < .001).

The spleen was predominantly pink‐red and this contrasted with its dark blue to red‐purple colour in healthy *C. taurus* and had dimensions similar to the pyloric stomach. The spleen's proportional length and diameter were significantly less than the respective means in healthy *C. taurus* both *p* < .001) and represented reductions of ~53% and ~67%, respectively. Finally, the pancreas was an abnormally pale cream colour, appeared markedly reduced in size, but was not measured or weighed for quantitative comparison.

On dissection, the GI tract was devoid of dietary items in the cardiac stomach, partially digested material in the pyloric stomach and chime in the intestine. The cardiac stomach lumen was markedly reduced and contained ~5 ml of clear, aqueous fluid. The pyloric stomach had a similar appearance, but minimal fluid was evident. The normal pale green chime was completely absent from the intestine and replaced by ~5 ml of pink, aqueous fluid. Distal to the orange fishing line, the mucosa and columnar spiral valve were clean and pale pink (Figure 1d). Combined, these observations suggested a complete stasis of the GI tract.

The suspected hook formed the nidus of the intestinal HSE that was cream in colour and had a rough surface (Figure 1d). The point of a hook protruded through the enterolith and the nylon fishing line attached to the HSE exited through the perforation the intestinal wall (Figure 1c). The HSE was located entirely within the confines of the rings of the columnar spiral valve where it occluded approximately 60% of the intestinal lumen. Additionally, the mucosa and submucosa of the spiral valve rings exhibited a dark blue discolouration immediately proximal to the enterolith (Figure 1d).

Finally, the sexually immature status indicated by the TL and PPS was confirmed by the limited development of the ovary and anterior reproductive tract, no posterior expansion of the thin‐walled uteri (~10 mm in diameter) and the presence of uterine hymen.

### 3.3 Weight, composition, mineralogy and structure of the HSE

**FIGURE 2:**
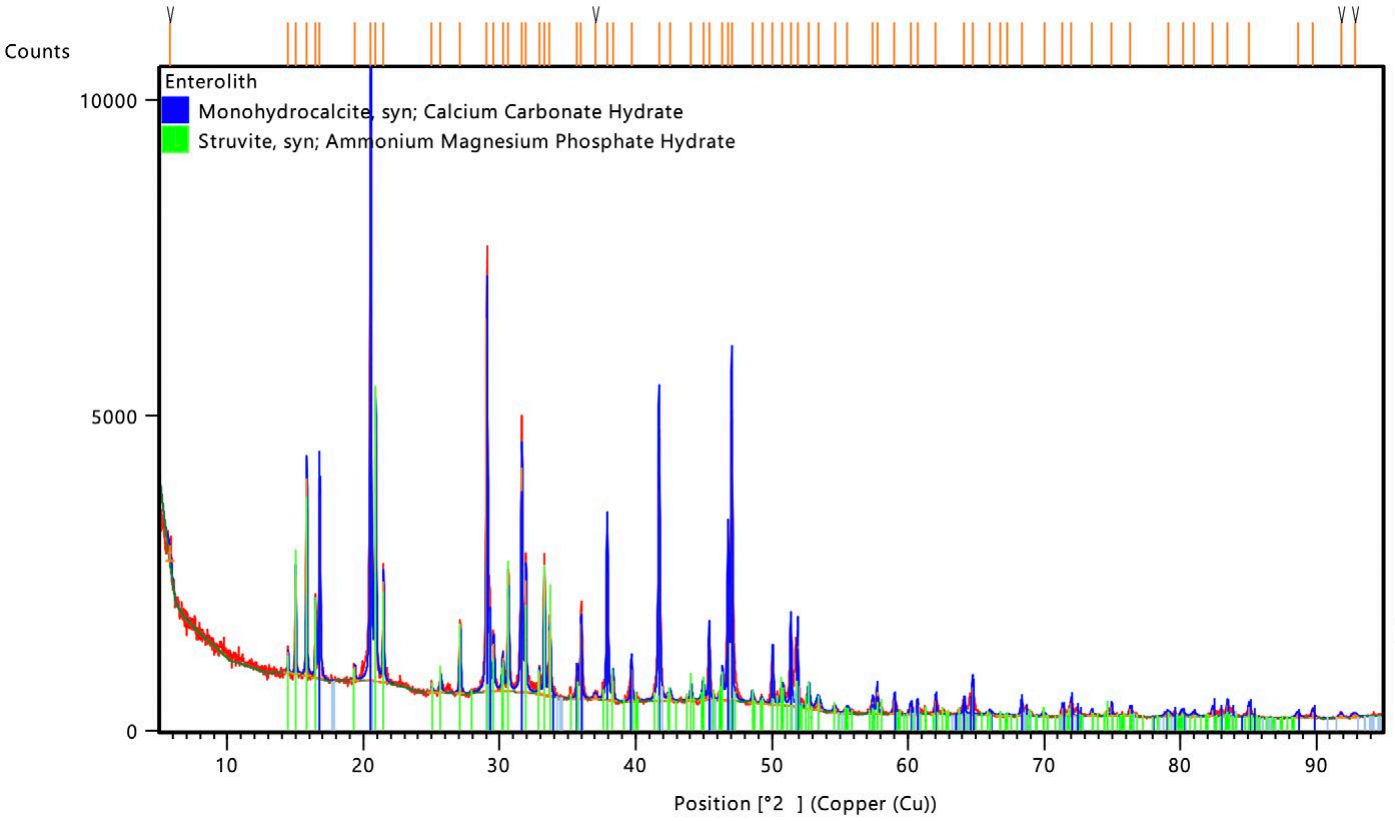
X‐ray diffraction patterns attributable to monohydrocalcite and struvite obtained from the hook‐shaped enterolith removed from the intestine of the stranded, juvenile female grey nurse shark (*Carcharias taurus*) recovered from One Mile Beach, NSW, Australia

The dry weight of HSE and attached nylon line was 20.5 g. Elemental analyses revealed the major elements calcium (~26 wt%) and phosphorus (20 wt%), with minor elements sulphur, chlorine and potassium (0.1 to 1 wt%), and trace elements iron, nickel, copper, zinc and strontium (0.01–0.1 wt%). All other elements (heavier than silicon) were present at concentrations of <0.01 wt%. X‐ray diffractometry showed that the HSE was predominantly (~70 wt%) comprised of the mineral monohydrocalcite (Ca[CO_3_].[H_2_O]) and a lesser amount (~30 wt%) of the mineral struvite (Mg [NH_4_] [PO_4_]. [H_2_O]_6_; Figure 2).

**FIGURE 3:**
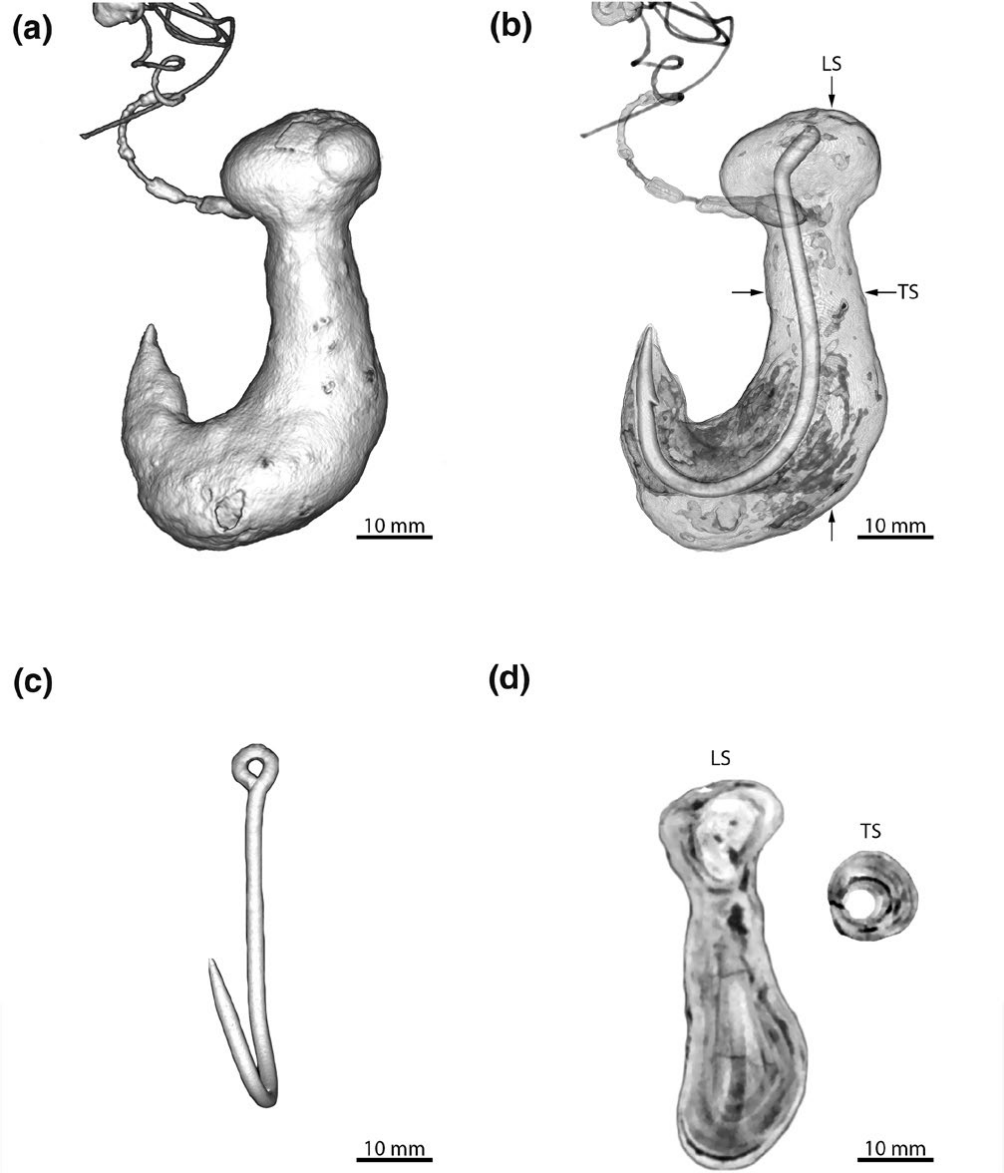
Results of CT scan of hook‐shaped enterolith from the stranded, juvenile female grey nurse shark (*Carcharias taurus*) recovered from One Mile Beach, NSW, Australia showing: (a) three‐dimensional, hook‐shaped external appearance of the enterolith with attached nylon line; (b) three‐dimensional internal appearance with hook nidus and arrows denoting sections in Figure 3d; (c) offset J‐hook with upturned ring eye showing offset; and (d) longitudinal (LS) and transverse (TS) sections of the enterolith taken at the points indicated by the arrows in Figure 3b

The CT scan clearly defined the three‐dimensional, external and internal features of the enterolith which were centred on the hook and led to its particular shape (Figure 3a and b). The HSE had a total length of 58 mm and maximum diameters of 8, 19 and 21 mm, respectively, just below the barb, around the shank, and in the bulbous region around the eye of the hook. The hook itself was offset, had an upturned ring eye (with attached nylon line), wire diameter of 2 mm, a total length of 50 mm, a straight shank of 30 mm, a bite length of 20 mm and a gape of 20 mm (Figure 3c). Combined, these dimensions suggested that the hook was most likely a 7/o offset J‐hook. The mean weight of 20 new offset J‐hooks of a similar size, shape and wire diameter (Owner Hooks, Model No. 5115‐171) was 1.70 g (*SD*, 0.06 g). This indicated that the weights of the actual hook (nidus) and HSE would have been ~2 g and ~18 g, respectively. Finally, transverse and longitudinal sections of the CT scan (Figure 3d) showed primary, concentric lamellae (concretions) around the hook nidus and provided an obvious history of growth, but the period of time over which the enterolith was laid down could not be estimated.

## 4 DISCUSSION

While this account is not the first description of an enterolith or urolith in *C. taurus*, it is to our knowledge, the first description of a hook‐shaped enterolith in any marine animal. Given the apparent GI tract stasis, it is very likely that this shark became anorexic and the minimal energy assimilation led to the 60% and 89% reductions in TW and LW, respectively. The advanced cachectic state of the shark also suggested that no demonstrable skeletal growth would have occurred after the initial hooking and enterolithiasis. This outcome is similar to the captive 2.70 m TL female *C. taurus* in London Aquarium that exhibited severe weight loss over 18 months (Monreal‐Pawlowsky et al., 2017; Thornton et al., 2012). At surgery, a 100 mm diameter spherical enterolith, causing partial obstruction of the spiral valve, was removed from the intestine. The enterolith had concentric lamellate layers and the nidus was an undigested octopus beak. Unfortunately, the shark died 7 hr after surgery without recovering from anaesthesia. The subsequent necropsy also noted a markedly reduced liver mass and suggested that this likely contributed to the animal's demise. Importantly, the cachectic changes present in both studies are similar to those in a blue shark with a retained fishing hook (Borucinska et al., 2001), and in humans and other wild/domesticated animals (Freeman, 2018).

### 4.1 Enterolithiasis

The CT scan of the HSE in the stranded, female *C. taurus* showed primary, concentric lamellae (concretions) around the hook nidus and these provided an obvious history of growth, but the period of time over which the enterolith was deposited could not be estimated. The HSE was comprised of the minerals monohydrocalcite and struvite. This contrasted markedly with the calcium hydroxyapatite enterolith from the captive *C. taurus* in the London aquarium (Monreal‐Pawlowsky et al., 2016; Thornton et al., 2012), the composition of which was attributed to fish bones from the shark's predominantly piscivorous diet. From a geological perspective, monohydrocalcite is a rare mineral, but has been documented naturally in saline lakes with temperatures ranging from 0 to 25°C in North America, Asia and Australasia (Fukushi, Munemoto, Sakai, & Yagi, 2011 and references therein). Its formation occurs with a pH exceeding 8.0, a magnesium/calcium ratio exceeding 4.0 (Fukushi et al., 2011) and can, via biochemical processes, form the mineral components of fish otoliths (Campana, 1999) and guinea pig uroliths (Hawkins, Ruby, Drazenovich, & Westropp, 2009). Interestingly, when sharks have been experimentally fasted, they continue to drink small quantities of seawater (Anderson, Taylor, Good, Hazon, & Grosell, 2007; Wood, Kajimura, Bucking, & Walsh, 2009) and this contributes, in part, to an increased magnesium/calcium ratio favouring the precipitation of monohydrocalcite and an alkaline intestinal environment (Anderson et al., 2007, 2010; Wood et al., 2009). To this end, seawater samples collected over 13 months (2006–2007) from waters off the New South Wales mid‐north coast in the shark's last perceived habitat consistently produced magnesium/calcium ratios exceeding 4.2 (Ellis & Otway, 2011). Additionally, an analysis of the gastrointestinal handling of water and solutes has shown that the bamboo shark, *Chiloscyllium plagiosum*, also maintains a magnesium/calcium ratio exceeding 10.0 in the chime of its intestine (Anderson et al., 2010). The absence of food items, GI tract stasis and the occurrence of monohydrocalcite in the HSE suggests a history of seawater drinking and anorexia in the stranded, female *C. taurus*. Moreover, the conspicuous absence of the mineral hydroxyapatite from the HSE in the stranded, female *C. taurus* can be argued as further evidence of anorexia in this shark.

Precipitation of struvite in gastrointestinal and urinary calculi is associated with an alkaline pH, the presence of phosphate ions, a super‐saturation of magnesium ions and the presence of ammonium ions that are often generated via bacterial hydrolysis of urea (Hassel et al., 2001; Rouff, Lager, Arrue, & Jaynes, 2018). Struvite is commonly found in enteroliths of equids (e.g. Hassel et al., 2001) but has also been found in marine mammals (Burdett & Osborne, 2010) and in the uroliths of various animals (e.g. Domingo‐Neumann, Ruby, Ling, Schiffman, & Johnson, 1996; Houston, Moore, Favrin, & Hoff, 2004). Canine and feline uroliths are often associated with infections caused by urease positive bacteria with the precipitation of struvite occurring together with apatite (Le Geros & Le Geros, 1984). Moreover, the predominant mineral deposited is ultimately determined by the urinary magnesium/calcium ratio with struvite favoured by greater magnesium concentrations (Buffington, 1994; Le Geros & Le Geros, 1984).

With this in mind, it was not surprising to find struvite present in the HSE from the stranded, female *C. taurus* given that sharks support a range of urease positive bacterial genera (e.g. *Vibrio, Pseudomonas, Aeromonas*) in their tissues including the GI tract, liver and kidneys, (Grimes, Brayton, Colwell, & Gruber, 1985; Knight, Grimes, & Colwell, 1988; Smith, 1992). Additionally, phosphate ions have been demonstrated to increase in concentration with fasting in the dogfish, *Squalus acanthias*, (Martini, 1978). The alkaline intestinal environment and increased magnesium concentrations (likely derived from seawater drinking) would have also contributed to enterolithiasis with struvite deposition.

While enteroliths containing struvite have not previously been observed in *C. taurus*, struvite uroliths have been found in a captive 70 kg female *C. taurus* held at Sea World, Florida USA (Walsh & Murru, 1987). The captive shark exhibited a small, but stable, appetite over 3.5 years followed by acute anorexia and sudden death in spite of veterinary intervention. At necropsy, two uroliths with a combined weight of 9.0 g and composed of struvite (80%) and carbonated hydroxyapatite (15%) were found incidentally in the urogenital sinus. While the cause of death was inconclusive, it is likely that a bacterial infection contributed to the shark's demise as struvite uroliths are often associated with urinary tract infections in a range of terrestrial animals (Hassel et al., 2001; Rouff et al., 2018).

### 4.2 Hook migration

There are at least two possible hook migration routes from the cardiac stomach to the intestine. First, the hook could have passed through the pyloric stomach and then entered the intestine without becoming embedded in the mucosa. Second and more likely, the hook perforated the cardiac stomach wall and this was followed soon after by perforation of the intestinal wall. This parsimonious sequence of events would have been facilitated by the initial (normal) apposition of the cardiac stomach and intestine. The gut contents of healthy *C. taurus* provided further evidence with many individuals having fish with embedded hooks attached to fishing line (100–200 mm long) in the cardiac stomach lumen. As sharks use the lateral line and electroreception to locate and capture prey (Kalmijn, 1971; Maruska, 2001), it is very likely that the fish were consumed following attraction to vibrations caused by the erratic movements of the hooked fish struggling on an angler's line. With this in mind, the presence of the 120‐mm‐long nylon fishing line attached to the hook nidus within the enterolith provides strong, correlative evidence for the same process occurring with the stranded, female *C. taurus*. Hence, we hypothesize that this shark took a fish on an angler's line and following consumption/digestion, the hook was free to move within the cardiac stomach lumen and subsequently settled to the ventral, mucosal wall. With continued feeding and muscular movements, pressure on the hook caused it to perforate the cardiac stomach wall and then the thin, muscular wall of the sub‐adjacent intestine. Serendipitously, the hook perforated the intestine where the posterior intestinal artery joins and hook hypomotility was assured on entering lumen between the rings of the columnar spiral valve with additional anchoring provided by the irregularly coiled fishing line protruding through the intestinal wall and into the abdominal cavity. While the specific pathogenesis of enterolithiasis remains unknown (Hassel et al., 2001), it is likely that formation of the HSE would have commenced at some stage thereafter as the other contributing factors developed within the intestine. Additionally, the intestinal perforation at the merger of the posterior intestinal artery would also have facilitated the invasion of GI tract bacteria into the systemic circulation and abdominal cavity leading to infection and disease in line with previous studies (Borucinska et al., 2001, 2002, 2003).

## 5 SUMMARY AND CONCLUSION

The precise cause of death of the stranded, female *C. taurus* could not be established because the degree of *post‐mortem* autolysis prevented detailed histopathological analyses. Nevertheless, at necropsy the shark exhibited advanced cachexia with severe loss of muscle and liver mass combined with significant reductions in the size of the GI tract organs and alteration to their normal positions within the abdominal cavity. Migration of the retained hook from the cardiac stomach to the intestinal spiral valve likely led to anorexia and a chronic bacterial infection. After development of appropriate conditions, enterolithiasis would have ensued with the formation of the monohydrocalcite/struvite enterolith around the offset, 7/o J‐hook. Ongoing anorexia, over the indeterminable duration of enterolithiasis, would have further exacerbated the loss of muscle and liver mass producing the advanced cachectic condition. To this end, the aetiology appeared similar to previously published accounts of enterolithiasis and urolithiasis in *C. taurus*. Irrespective of this, however, retention of the hook in the GI tract ultimately led to the shark's mortality, an outcome that was also consistent with previous studies.

## CONFLICTS OF INTEREST

The authors declare that they have no conflicts of interest.

## AUTHOR CONTRIBUTION

**Nicholas Mark Otway:** Conceptualization; Data curation; Formal analysis; Funding acquisition; Investigation; Methodology; Project administration; Resources; Validation; Visualization; Writing‐original draft; Writing‐review & editing. **Greg J West:** Data curation; Formal analysis; Investigation; Methodology; Resources; Software; Validation; Visualization; Writing‐original draft; Writing‐review & editing. **Damian B Gore:** Data curation; Formal analysis; Investigation; Methodology; Resources; Software; Validation; Visualization; Writing‐original draft; Writing‐review & editing. **Jane E Williamson:** Data curation; Formal analysis; Investigation; Methodology; Resources; Validation; Visualization; Writing‐original draft; Writing‐review & editing. NMO initiated the work and was responsible for the experimental design of this case report. NMO, GJW, DBG and JEW all contributed to data collection and analysis, and the writing/editing of the manuscript.

## ETHICAL STATEMENT

The study was carried out under an Animal Research Authority (99/14—Port Stephens) from the NSW Department of Primary Industries (Fisheries NSW) Animal Care and Ethics Committee issued in accordance with the National Health and Medical Research Council Australian code of practice for the care and use of animals for scientific purposes (8th Edition, 2013). The overarching research project and its associated sampling protocols were done under a scientific research permit (Permit No. P01/0059[A]) issued by the NSW Department of Primary Industries (Fisheries NSW).

The authors confirm that the ethical policies of the journal, as noted on the journal's author guidelines page, have been adhered to.

### PEER REVIEW

The peer review history for this article is available at https://publons.com/publon/10.1002/vms3.333.
